# Ventricular Tachycardia in a Pediatric Patient with High-Risk Thrombotic Thrombocytopenia Purpura

**DOI:** 10.1155/2023/6466680

**Published:** 2023-01-18

**Authors:** Taylor J. Kratochvil, Jeffrey A. Robinson

**Affiliations:** ^1^Department of Pediatrics, Boston Children's Hospital and Harvard Medical School, Boston, MA, USA; ^2^Department of Pediatrics, Boston Medical Center and Boston University School of Medicine, Boston, MA, USA; ^3^College of Medicine, University of Nebraska Medical Center, Omaha, NE, USA; ^4^Division of Cardiology, Department of Pediatrics, University of Nebraska Medical Center, Omaha, NE, USA; ^5^The Dr. C.C. and Mabel L. Criss Heart Center, Children's Hospital and Medical Center, Omaha, NE, USA

## Abstract

An 8-year-old previously healthy male was diagnosed with thrombotic thrombocytopenic purpura (TTP) and increased serum cardiac troponin I. Telemetry recorded non-sustained ventricular tachycardia, without ST-segment changes or other abnormalities on serial electrocardiogram. This case illustrates that cardiac monitoring by telemetry should be considered in high-risk TTP with elevated cardiac troponin.

## 1. Introduction

Thrombotic thrombocytopenic purpura (TTP) is caused by a functional deficiency of a disintegrin and metalloprotease with thrombospondin type 1 repeats, member 13 (ADAMTS13). ADAMTS13 is a plasma protease responsible for proteolysis of von Willebrand factor (VWF). In its absence, uncleaved VWF multimers circulate in the blood, resulting in platelet binding and increased thrombotic risk [1].

While *hereditary TTP* arises from biallelic ADAMTS13 mutations, *acquired TTP* is caused by autoantibodies increasing the rate of ADAMTS13 elimination or inhibiting its function [1]. It is estimated that only ~10% of all TTP cases occur in childhood [2], with less than one case per million children per year [3, 4], and is particularly rare in children younger than 9 years old [1].

## 2. Case Presentation

An 8-year-old Burmese male presented to the emergency department with a 7-day history of dizziness, jaundice, and worsening ecchymoses/petechiae on the extremities and trunk. This was preceded by 2 months of fatigue and nausea, without diarrhea. Ecchymoses gradually increased in number and size, without history of trauma.

The patient was born at term in a resource-limited Thai refugee camp, and spent 1 month in the hospital for neonatal feeding support and weight gain. At 7 years of age (8 months prior to hospital presentation), the patient immigrated with his immediate family to the United States. Thrombocytopenia (platelet count: 120 × 10^3^/*μ*L) and borderline anemia (hemoglobin: 11.9 g/dL) were noted at a refugee health exam, with negative screening for hepatitis B, human immunodeficiency virus, and tuberculosis. Neither the patient nor his family members have experienced similar symptoms.

The differential diagnosis for this patient included pathologies that manifest as or mimic a coagulopathy. These included severe nutritional deficiencies (e.g., iron, vitamin B12, and folate), systemic disease (e.g., hepatitis, hemolytic uremic syndrome, and disseminated intravascular coagulopathy), blood dyscrasias (e.g., leukemia, idiopathic thrombocytopenic purpura, and inherited/acquired TTP), and non-accidental trauma.

Initial values (Table 1) demonstrated thrombocytopenia, anemia, an elevated reticulocyte count, and a peripheral smear positive for schistocytes and spherocytes. Coagulation studies were normal with an elevated D-dimer. A comprehensive metabolic panel was normal for age and indicated normal kidney function. Coombs' test was negative. The patient was admitted to the hospital, where a low ADAMTS13 (<5%), and a positive inhibitor antibody confirmed the diagnosis of acquired TTP.

In consultation with the hospital's hematology team, the patient received a unit of packed red blood cells and platelets prior to the urgent placement of an internal jugular pheresis catheter for daily plasma exchange therapy (PLASMIC score of 7) [5]. In addition, the patient was started on prednisolone (0.5 mg/kg by mouth, twice daily) and rituximab (375 mg/m^2^ intravenous, once weekly for four doses).

**Figure 1:**
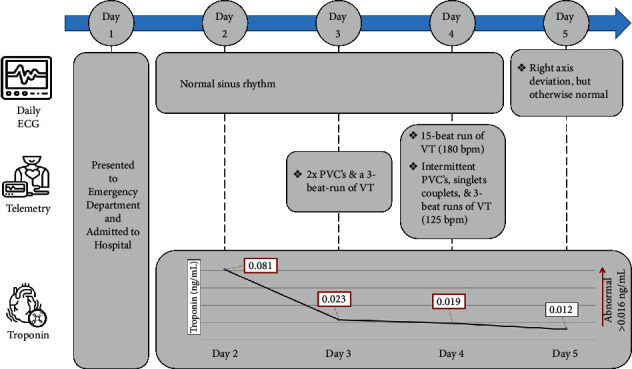
Cardiac monitoring and troponin inpatient cardiac monitoring, with time measured from the patient's initial hospital presentation. PVC = premature ventricular contraction; VT = ventricular tachycardia.

As TTP's microangiopathy can manifest in multiorgan damage, cardiac monitoring was initiated. The patient was found to have an elevated cardiac troponin I (peak: 0.081 ng/mL; reference range [RR]: ≤0.016 ng/mL) and intermittent ventricular tachycardia (VT)/premature ventricular contractions (Figure 1). On day 4 of admission, there was an asymptomatic 15-beat run of monomorphic VT at rest, with the shortest R–R cycle length of 330 ms.

Serum electrolytes were monitored via serial comprehensive metabolic panels, which demonstrated hypocalcemia (total serum calcium 8.6 mg/dL; ionized calcium 1.20 mmol/L). All other electrolytes were within normal limits for age. Additionally, a screening echocardiogram demonstrated normal segmental cardiac anatomy and normal biventricular size and systolic function with an anomalous origin of the right coronary artery from the left sinus of Valsalva and a small patent foramen ovale with left-to-right flow. Due to the patient's elevated cardiac troponin, prophylactic heparin was initiated and was transitioned to aspirin for oral anti-platelet therapy upon resolution of the cardiac troponinemia.

To evaluate for a potential rheumatologic etiology of the patient's symptoms, further investigation found a positive antinuclear antibody (ANA), positive anti-double stranded DNA (anti-dsDNA) antibodies, positive SSA 60, and a low C3 but normal C4. Additional lab tests demonstrated no renal (negative urinalysis and urine protein/creatinine ratio within normal limits), respiratory (pulmonary function test with spirometry, lung volumes, and diffusion capacity within normal limits), or neurovascular (unremarkable magnetic resonance imaging/magnetic resonance angiography of the brain) abnormalities. With these findings, the patient was diagnosed with systemic lupus erythematosus and was started on pulse-dosed steroids with solumedrol 30 mg/kg/day for three doses, followed by a 3-day course of high-dose, intravenous methylprednisolone 30 mg/kg/day.

Troponin levels were trended and returned to normal on day 4 of admission. Improvement in troponin levels were likely multifactorial in the setting of the patient's pharmacologic and plasma exchange treatments. Prior to hospital discharge on day 16 of admission, cardiac magnetic resonance imaging (MRI) showed no evidence of delayed gadolinium enhancement, but confirmed the anomalous origin of the right coronary artery from the left sinus of Valsalva. The patient was discharged from hospital to home and prescribed aspirin, prednisolone, nizatidine, ondansetron, and acetaminophen.

The patient was scheduled for a 6-month outpatient follow-up appointment with a pediatric cardiac electrophysiologist. The patient reported no symptoms, and a Holter monitor at 6 months demonstrated sinus rhythm throughout, without evidence of ectopy or ventricular arrhythmia. Due to the patient's temporarily elevated troponin, inpatient electrophysiological derangements, and the abnormal coronary artery anatomy, he will require ongoing evaluation with a pediatric cardiologist, sequential transthoracic echocardiograms, and exercise stress testing [6]. This patient was diagnosed with systemic lupus erythematosus following the acute presentation with TTP, which is a known association in pediatric patients [7].

## 3. Discussion

Despite negative serial electrocardiograms (ECGs), this patient's asymptomatic non-sustained VT was only discovered because he was being monitored on telemetry. This illustrates the importance of monitoring cardiac troponin as a trigger for further cardiac monitoring. Adult treatment guidelines specify screening for microangiopathy and coronary artery involvement with serum cardiac troponin and ECG at the time of TTP diagnosis [8]. Though pediatric guidelines suggest cardiac troponin should be measured in patients with TTP, this may not occur due to a perceived rarity of microvascular thrombi and cardiac involvement in the children [7, 9]. In adult populations, ischemic myocardial injury has been identified postmortem as the cause of death in patients with TTP [10]. This should be considered for pediatric patients with an acute episode of acquired TTP.

Additionally, this case suggests that patients with high-risk TTP (elevated cardiac troponin) should receive cardiac monitoring by telemetry. Screening for cardiac microvascular thrombosis in patients is important, as elevated cardiac troponin is an independent risk factor for mortality in patients with TTP [11]. Monitoring for arrhythmias in patients with TTP is important, as patients who develop acute myocardial ischemia may present with atrial fibrillation, atrial flutter, supraventricular tachycardia, or congestive heart failure [12].

It is possible that the patient's elevated troponin and electrophysiological derangements were secondary to other coexisting conditions, though the authors suspect his cardiac pathology is primarily a result of his TTP and subsequent physiologic stress on cardiac myocytes. While autoimmune rheumatologic diseases may present with electrophysiological changes, systemic lupus erythematosus is more frequently associated with sinus tachycardia, atrial fibrillation, and atrial ectopic beats [13]. Furthermore, the patient's anomalous origin of the right coronary artery from the left sinus of Valsalva was not associated with an intramural course or ostial stenosis. This is believed to be low-risk for symptoms, troponinemia, arrhythmia, and ischemia [14].

## 4. Conclusions

This case emphasizes the importance of screening pediatric patients diagnosed with TTP for elevated cardiac troponin I. If the patient is determined to be high-risk via an elevated cardiac troponin I, subsequent telemetric monitoring during their acute illness course is important to screen for electrophysiological derangements.

## Figures and Tables

**Table 1:** Admission laboratory data.

	Lab value	Reference range
Hgb, g/dL	6.0	11.0–13.3
Platelet count, ×10^3^/*μ*L	5	150–400
ARC, ×10^6^/*μ*L	0.209	0.030–0.110
WBC, ×10^3^/*μ*L	6.59	4.5–10.5
PTT, seconds	32	24–35
PT, seconds	13.0	9.8–12.6
INR	1.1	N/A
D-dimer, ng/mL	1340	≤500

ARC = absolute reticulocyte count; Hgb = hemoglobin; INR = international normalized ratio; PT = prothrombin time; PTT = partial thromboplastin time; WBC = white blood cell count.

## Data Availability

The data analyzed in this study are will not be publicly released in accordance with HIPAA guidelines.

## Abbreviations

ADAMTS13: A disintegrin and metalloprotease with thrombospondin type 1 repeats, member 13
ECG: Electrocardiogram
Hgb: Hemoglobin
INR: International normalize ratio
PT: Prothrombin time
PTT: Partial thromboplastin time
RR: Reference range
TTP: Thrombotic thrombocytopenic purpura
VT: Ventricular tachycardia
WBC: White blood cell count.
